# Persistence and Intra-Host Genetic Evolution of Zika Virus Infection in Symptomatic Adults: A Special View in the Male Reproductive System

**DOI:** 10.3390/v10110615

**Published:** 2018-11-07

**Authors:** Danielle B. L. Oliveira, Giuliana S. Durigon, Érica A. Mendes, Jason T. Ladner, Robert Andreata-Santos, Danielle B. Araujo, Viviane F. Botosso, Nicholas D. Paola, Daniel F. L. Neto, Marielton P. Cunha, Carla T. Braconi, Rúbens P. S. Alves, Monica R. Jesus, Lennon R. Pereira, Stella R. Melo, Flávio S. Mesquita, Vanessa B. Silveira, Luciano M. Thomazelli, Silvana R. Favoretto, Franciane B. Almonfrey, Regina C. R. M. Abdulkader, Joel M. Gabrili, Denise V. Tambourgi, Sérgio F. Oliveira, Karla Prieto, Michael R. Wiley, Luís C. S. Ferreira, Marcos V. Silva, Gustavo F. Palacios, Paolo M. A. Zanotto, Edison L. Durigon

**Affiliations:** 1 Department of Microbiology, Institute of Biomedical Sciences, University of São Paulo, São Paulo, SP 05508-000, Brazil; danibruna@gmail.com (D.B.L.O.); ericaarmendes@gmail.com (É.A.M.); robert_andreata@hotmail.com (R.A.-S.); daniellebastos@yahoo.com.br (D.B.A.); nicholasdipaola@gmail.com (N.D.P.); danielviro@gmail.com (D.F.L.N.); marieltondospassos@gmail.com (M.P.C.); cabraconi@gmail.com (C.T.B.); rubens.bmc@gmail.com (R.P.S.A.); modrigues4@gmail.com (M.R.J.); lennon_rp@hotmail.com (L.R.P.); stellmelo@gmail.com (S.R.M.); flavio.mesquita@usp.br (F.S.M.); vanessa.silveirabio@gmail.com (V.B.S.); lucmt@usp.br (L.M.T.); lcsf@usp.br (L.C.S.F.); pzanotto@usp.br (P.M.A.Z.); 2 Medical School Clinic Hospital, University of São Paulo, São Paulo, SP 05403-000, Brazil; giuliana.durigon@gmail.com (G.S.D.); fran_almonfrey@hotmail.com (F.B.A.); kader@usp.br (R.C.R.M.A.); 3 Center for Genome Sciences, US Army Medical Research Institute of Infectious Diseases, Frederick, MD 21702, USA; jtladner@gmail.com (J.T.L.); karla.prieto.ctr@mail.mil (K.P.); michael.r.wiley19.ctr@mail.mil (M.R.W.); gustavo.f.palacios.ctr@mail.mil (G.F.P.); 4 The Pathogen and Microbiome Institute, Northern Arizona University, Flagstaff, AZ 86011-4073, USA; 5 Virology Laboratory, Butantan Institute, São Paulo, SP 05503-900, Brazil; viviane.botosso@butantan.gov.br (V.F.B.); joel.megalegabrili@gmail.com (J.M.G.); denise.tambourgi@butantan.gov.br (D.V.T.); 6 Pasteur Institute, State Health Department, São Paulo, SP 1103-000, Brazil; srfavoretto@usp.br; 7 Immunochemistry Laboratory, Butantan Institute, São Paulo, SP 05503-900, Brazil; 8 Department of Cellular and Developmental Biology, Institute of Biomedical Sciences, University of São Paulo, São Paulo, SP 05508-000, Brazil; sfolivei@gmail.com; 9 Department of Environmental, Agricultural and Occupational Health, University of Nebraska Medical Center, Omaha, NE 68198-4388, USA; 10 Institute of Infectology Emílio Ribas e Pontifícia Universidade Católica (PUC-SP), São Paulo, SP 01246-900, Brazil; mvsilva@pucsp.br

**Keywords:** Zika virus, flavivirus, arbovirus, sexual transmission, host genetic variation, immune response

## Abstract

We followed the presence of Zika virus (ZIKV) in four healthy adults (two men and two women), for periods ranging from 78 to 298 days post symptom onset. The patients were evaluated regarding the presence of the virus in different body fluids (blood, saliva, urine and semen), development of immune responses (including antibodies, cytokines and chemokines), and virus genetic variation within samples collected from semen and urine during the infection course. The analysis was focused primarily on the two male patients who shed the virus for up to 158 days after the initial symptoms. ZIKV particles were detected in the spermatozoa cytoplasm and flagella, in immature sperm cells and could also be isolated from semen in cell culture, confirming that the virus is able to preserve integrity and infectivity during replication in the male reproductive system (MRS). Despite the damage caused by ZIKV infection within the MRS, our data showed that ZIKV infection did not result in infertility at least in one of the male patients. This patient was able to conceive a child after the infection. We also detected alterations in the male genital cytokine milieu, which could play an important role in the replication and transmission of the virus which could considerably increase the risk of ZIKV sexual spread. In addition, full genome ZIKV sequences were obtained from several samples (mainly semen), which allowed us to monitor the evolution of the virus within a patient during the infection course. We observed genetic changes over time in consensus sequences and lower frequency intra-host single nucleotide variants (iSNV), that suggested independent compartmentalization of ZIKV populations in the reproductive and urinary systems. Altogether, the present observations confirm the risks associated with the long-term replication and shedding of ZIKV in the MRS and help to elucidate patterns of intra-host genetic evolution during long term replication of the virus.

## 1. Introduction

Zika virus (ZIKV) was first identified in 1947 in Africa and subsequently reached Asia and, more recently, the Americas [1,2]. Today, more than 80 countries around the globe have reported cases of active ZIKV transmission and the recent outbreaks have associated the virus with several neurological disorders, including Guillain-Barré syndrome and congenital neurologic birth defects [3,4,5,6]. Due to its rapid spread, there is a need to understand how the virus interacts with the human host which includes persistence and shedding in biological fluids in order to improve diagnosis, prevention and treatment.

Reports have shown that ZIKV RNA is present in several body fluids, including urine, blood, saliva, breast milk and secretions from the vaginal tract [7,8,9,10], being cleared early from serum but still detected in whole blood, saliva and urine for more than two weeks after symptoms onset [11,12,13,14]. ZIKV can also replicate and persist in the male reproductive system and ZIKV RNA has been detected within semen for up to 6 months after initial infection in the male reproductive system (MRS) [15,16]. The potential for persistent ZIKV infections in the MRS raises concerns ranging from the demonstrated sexual transmission of the virus to the damage of germ cells and generation of poor-quality sperm. In fact, ZIKV infection has been shown to cause infertility in mice [17,18]. In addition, since the ZIKV genome is RNA-based, the occurrence of spontaneous mutations may lead to the accumulation of genetic variants during persistent infection, particularly at the MRS, with unpredicted outcomes for the virus–host interactions.

In the present study, we followed the natural course of ZIKV infection in four symptomatic adults (two men and two women) infected with ZIKV in Brazil. The patients were monitored for the presence of the virus in different body fluids, including blood, saliva, urine and semen and for the development of an associated immune response, with emphasis on two male patients who shed the virus for up to 158 days after the initial symptoms. The presence of virus within semen samples, capable of replicating in cell cultures, allowed us to obtain almost full ZIKV genome sequences and evaluate the intra-host virus genetic evolution. These results led us to suggest that ZIKV shows an independent compartmentalization in MRS that may impact the fate of the virus and its interaction with the human host. In addition, we detected alterations in the genital cytokine milieu and a lack of detectable proinflammatory cytokines/chemokines in the blood.

## 2. Materials and Methods

### 2.1. Case Definition and Sample Collection

The cases were defined as individuals with ZIKV-like illness (acute onset of rash associated with at least one of the following symptoms: fever, conjunctivitis, pruritus or arthralgia) and the presence of ZIKV RNA in at least one of these clinical samples: serum, saliva, urine and semen. During the outbreak of ZIKV in Brazil, 117 patients with ZIKV illness were tested at the Clinical and Molecular Virology Laboratory in the Institute of Biomedical Sciences at University of São Paulo—USP, São Paulo, Brazil. From the six qRT-PCR positive patients, four of them accepted to be included in this study: ZIKV01, ZIKV17, ZIKV18 and ZIKV19. The study protocol was approved by the Ethics Committee on Research with Human Beings at the University of São Paulo and also evaluated and determined to be exempt by the US Army Medical Research Institute of Infectious Diseases (USAMRIID) Office of Human Use and Ethics. All four patients provided their informed consent for the use of their samples in this study. Following the initial positive result by qRT-PCR, all patients were tested weekly for the presence of ZIKV RNA in urine, serum, saliva and semen (for male patients), until all samples tested negative in at least two consecutive visits. Each patient had the first sample collected between days 3 and 7 after the onset of symptoms and the final sample collected up to a minimum of 78 days and a maximum of 235 days later (ZIKV01, ZIKV17, ZIKV18 and ZIKV19 for 78, 235, 119 and 155 days, respectively). The study timeframe for patient ZIKV01 was between February/2016–May/2016, for patient ZIKV17 between March/2016 until January/2017 and from April/2016 until September/2016 for patients ZIKV18 and ZIKV19.

#### Clinical Case

**Case-ZIKV01** is a 32-year-old woman who presented with a maculopapular rash on her trunk and upper limbs associated with abdominal pain and diarrhea. Two to four days after symptom onset, the exanthema became pruriginous and reached the lower limbs with the appearance of petechiae. She experienced intense epigastralgia that resolved after the fifth day. Arthralgia on ankles and edema of fingers were noticed on day 4 and lasted until day 7. No other symptoms, including fever, were reported. No significant laboratory findings were noted. The patient was subsequently followed for 78 days with no additional complications. The patient, who lives in São Paulo state, reported a trip to Espirito Santo state two weeks before the onset of symptoms.

**Case-ZIKV17** is a 33-year-old man who initially reported a fever lasting 2 days, followed by headache and retro-orbital pain (days 2–3). On day 4 he reported a maculopapular rash accompanied by intense itching and diffuse arthralgia. All symptoms cleared up by day 5. The patient was subsequently studied for 298 days. He experienced pain and edema of his right testicle at 25 days post-symptomatic onset. Orchitis due to ZIKV infection was diagnosed after confirmation through serology and qRT-PCR. No other potential agents were identified.

Further monitoring of ZIKV shedding was conducted, with express patient consent, since he was planning to conceive a baby with his partner. During the study period, the patient underwent two prostate ultrasounds (USG prostate) and two spermograms. The patient presented with prostatitis at 71 days post symptoms onset, which normalized by 218 days post symptoms onset. The spermograms were normal at days 100 and 223 post symptoms onset. The couple adopted strict preventive measures with daily use of insect repellent and barrier contraception during sexual intercourse for the entire period of confirmed virus shedding. After the first negative semen sample (day 168), the patient provided weekly samples (day 174) for 2 consecutive weeks, followed by two more biweekly collections (day 200) and finally a monthly collection (day 235), totaling 67 days with negative qRT-PCR results for all tested samples. At 235 days post symptoms onset the patient and his wife began actively trying to conceive. After two months (day 298) the patient’s wife confirmed pregnancy. At the time, and further during pregnancy, she was monitored for ZIKV infection by qRT-PCR, IgG and IgM. She remained negative.

**Cases-ZIKV18** and -**ZIKV19** are a husband and wife who experienced ZIKV-like illness in close succession. ZIKV19 is a 64-year-old man, who reported lumbar pain, fever and malaise at onset. After 3 days of symptoms a macular rash appeared which resolved after 6 days. The patient was subsequently studied for 155 days.

ZIKV18 is a 68-year-old woman who began experiencing symptoms of malaise five days after her husband. Three days after onset, a macular rash appeared on the face, body and limbs and erythematous plaque in the right leg, accompanied by fever, headache, ocular hyperemia and articular edema. On the fourth day the exanthema became more intense, followed by somnolence, chills and tiredness. The articular edema remained for 5 weeks. The patient was subsequently studied for 108 days. There were no other reports of complications during the study period.

**Figure 1:**
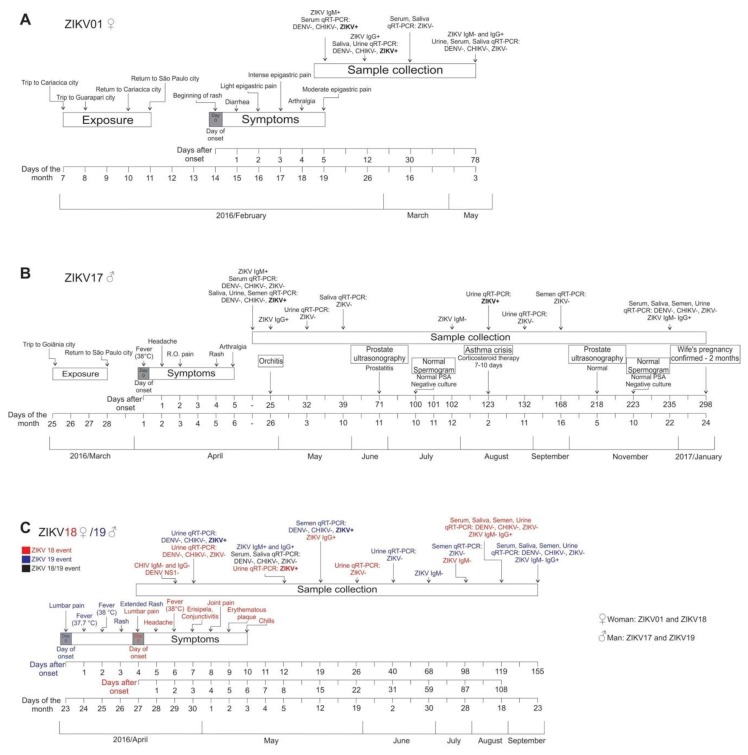
Timelines of ZIKV exposure, symptoms and sample collection in the four subjects enrolled in the present study. Periods of exposure, symptom onset, serology and molecular detection results of ZIKV in the followed subjects are described as: (**A**) case 1—female (ZIKV01), (**B**) case 2—male (ZIKV17), (**C**) case 3—female (ZIKV18) and case 4—male (ZIKV19). Day 0 denotes the onset of symptoms.

Timelines for all four patients with the principle clinical events and laboratorial results are shown in Figure 1.

### 2.2. Molecular and Classical Virology

For the serological analysis, ZIKV-specific IgG antibodies were measured using a nonstructural protein ∆NS1-ELISA [19] and ZIKV-specific IgM antibodies were detected using capture ELISA with a specific viral antigen for ZIKV [20]. For the molecular test, quantitative reverse transcription (qRT-PCR) TaqMan assays for detection of ZIKV, Dengue virus (DENV) and Chikungunya virus (CHIKV) RNA were performed. The assays, as well as the RNA extraction methods, are described in the Supplementary Appendix. To ensure RNA integrity and sample quality, all extracts were also tested for the presence of the human RNase P (RNP) gene by qRT-PCR. All extracts showed robust RNP cycle threshold (*Ct*) values ranging from 16.3 to *Ct* 33.5 (see Supplementary Appendix for details) demonstrating the integrity of the material collected. Assays for DENV [21] and CHIKV [22] were performed as controls for co-infection, as they have been reported in Brazil during the ZIKV outbreak. To evaluate infectivity, viral isolation was attempted for a subset of positive samples from the two male patients ZIKV17 and ZIKV19. For virus isolation, *Aedes albopictus* mosquito cells (C6/36) were inoculated with 500 µL of saliva, urine or semen. After 8–12 days of incubation, cells were collected and tested for ZIKV RNA presence by qRT-PCR. All samples were passaged in cell culture at least 3 consecutive times before being considered negative for ZIKV presence by qRT-PCR (for details see Supplementary Materials).

### 2.3. Virus Particle Detection in Semen by Immunofluorescence and Transmission Electron-Microscopy (IFA and TEM, Respectively)

The immunofluorescence assays were conducted using suspensions from semen samples collected on day 39 from patient ZIKV17 and day 40 from patient ZIKV19. The presence of ZIKV particles were also analyzed by TEM in semen from patient ZIKV17 (day 39) and in *Aedes albopictus* mosquito cells (C6/36) inoculated with the ZIKV17 semen sample. Thin sections were stained with uranyl acetate and lead citrate, and observed with a JEOL 1010 transmission electron microscope (for details see Supplementary Materials). C6/36 cells infected with ZIKV isolated from patient ZIKV17 were used as a positive control for both IFA and TEM assays.

### 2.4. Measurement of Cytokines and Chemokines in Serum and Seminal Plasma

The serum and semen samples from the male patients ZIKV17 and ZIKV19 were analyzed for the presence of the cytokines IL-17, IL-2, IL-4, IL-6, IL-10, TNF-α, IFN-γ and the chemokines CXCL8/IL-8, CCL5/RANTES, CXCL9/MIG, CCL2/MCP-1 and CXCL10/IP-10 using Cytometric Bead Arrays kits (Human Th1/Th2/Th17 and Human Chemokine from Becton Dickinson, CA, USA), according to the manufacturer’s instructions. The range of detection for cytokines was 20 to 5000 pg/mL and for chemokines was 10 to 2500 pg/mL. Samples were analyzed using a FACSCanto II flow cytometer, with FCAP Array 3.0 software, version 3.0, both from BD Biosciences. One-way ANOVA with Tukey post-tests were used to evaluate significant differences between groups. For correlation analyses, Pearson coefficient (r) was used. Statistical analysis was performed using GraphPad Prism software. Differences were considered statistically significant when p values were less than 0.05.

### 2.5. Genome Sequencing and Analysis

For samples that tested positive for ZIKV RNA by qRT-PCR, viral genetic diversity was characterized directly from the clinical specimens using next-generation sequencing. Sequencing libraries were prepared using the TruSeq RNA Access Library Prep kit (Illumina, Menlo Park, CA, USA) with custom ZIKV probes and sequenced using the MiSeq Reagent kit v3 (Illumina, Menlo Park, CA, USA) on an Illumina MiSeq (for details see supplementary appendix). Consensus-level ZIKV genome sequences were assembled for each sample using both de novo and reference-based approaches (for details see Supplementary Materials). These two approaches resulted in nearly identical sequences. However, for several lower coverage samples, contiguous assemblies could only be constructed with the reference-based approach. Consensus genomes were compared using median-joining haplotype networks (PopART v1.7.2) and an approximate maximum-likelihood phylogenetic reconstruction (FastTree v2.1.5). BEAST v1.8.3 [23] was used to estimate dN/dS and the rate of ZIKV evolution within the male reproductive system (MRS), HyPhy v2.3 was used to identify codons with evidence for positive, diversifying selection, and for samples with >50× average coverage, we examined intra host genetic variation using FreeBayes v1.0.2 [24] (for details see Supplementary Materials).

## 3. Results

### 3.1. Long Term Monitoring of Four Symptomatic ZIKV-Infected Patients

Four symptomatic ZIKV-infected individuals (two males; patients ZIKV17 and ZIKV19 with 33 and 64 years of age, respectively, and two females; patients ZIKV01 and ZIKV18 with 32 and 68 years) were followed for periods up to 298 days after the onset of symptoms (Figure 1). All patients became serological positive for ZIKV, both for IgM and IgG, during the course of the study. ZIKV IgM was detected in serum of patients ZIKV01, ZIKV17, ZIKV18 and ZIKV19 until day 78, 95, 59 and 54, respectively. All serum samples were positive for ZIKV IgG at the first time point tested, between 12 to 25 days after symptoms onset, and remained positive throughout the study (Figure 1).

**Figure 2:**
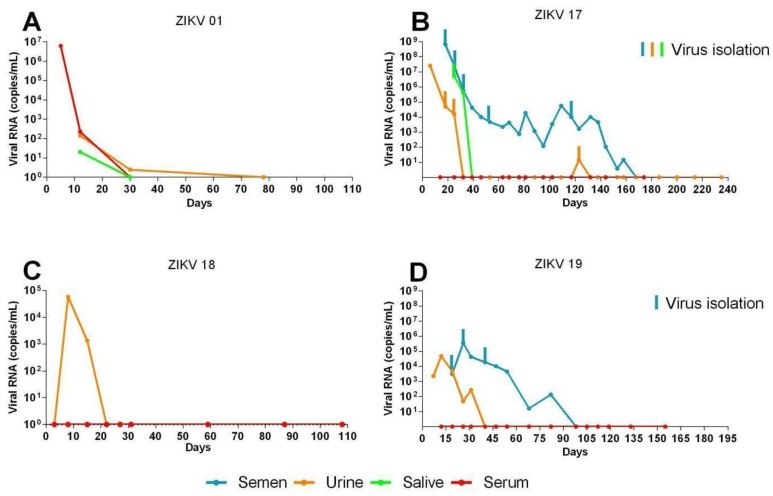
ZIKV RNA load in patient’s body fluids and clinical isolates on culture. The graph shows the viral load (genome copies/mL) versus excretion time (days after symptoms onset) of the weekly collection of urine (orange), saliva (green) and serum (red) samples from patients ZIKV01 (**A**), ZIKV17 (**B**), ZIKV18 (**C**) and ZIKV19 (**D**), in addition to the semen (blue) of the two men involved in the study (**C**,**D**). To confirm the viability of the excreted virus, the urine samples collected from days 18 and 25 after symptoms, saliva from day 25 and the semen from days 18, 25, 32, 53 and 117 for patient ZIKV17 and semen samples from patient ZIKV19 from days 19, 26 and 40 after symptoms onset were tested and the ones with positive results in cell culture are exhibited. All clinical samples and isolated samples were analyzed by qRT-PCR.

In the two females (ZIKV01 and ZIKV18) the ZIKA RNA was detected by qRT-PCR in urine, serum and saliva. ZIKV01 was the only patient in this study that developed viremia, which was detected at day 5 (6.2 × 10^7^ genome equivalents (ge)/mL) and lasted until day 12 (2.2 × 10^2^ ge/mL). ZIKV RNA was also detected in her saliva at day 12 (2 × 10^2^ ge/mL). Both patients had ZIKV RNA detected in urine samples from day 12 (10^2^ ge/mL) through day 30 (20 ge/mL) for patient ZIKV01 and at day 8 (5.7 × 10^4^ ge/mL) for patient ZIKV18 (Figure 2A,C).

**Figure 3:**
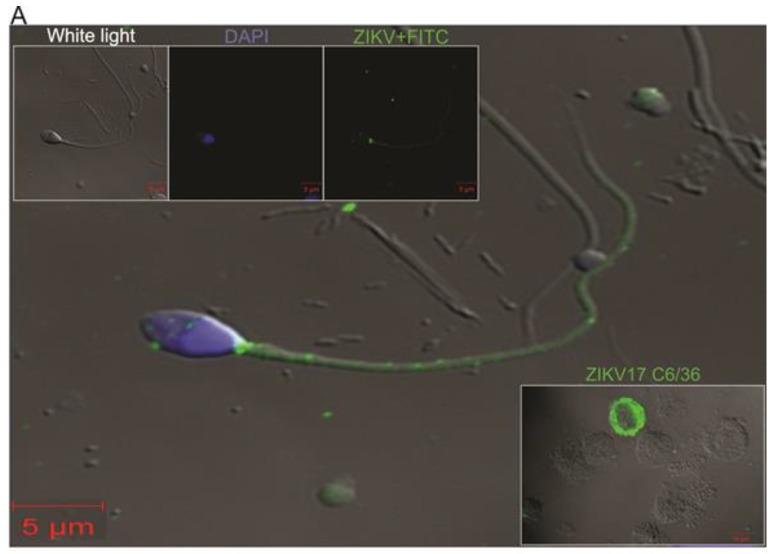

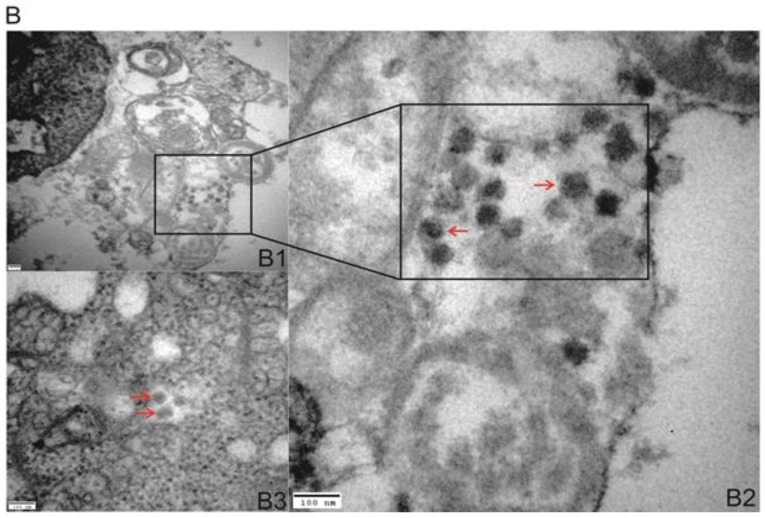
Detection of ZIKV in semen and C636 cells. (**A**) Detection by Indirect Immunofluorescence assay using anti-ZIKV specific antibody. Spermatozoa from semen sample collected from patient ZIKV17 at day 39 stained with FITC conjugate (in green) for virus location and with DAPI for nucleus staining (in blue). The viruses were located in the cytoplasm and flagella. C6/36 cell culture infected with virus from ZIKV17 semen sample. The cell infected presents with a green color (lower right panel). (**B**) Electron Microscopy of ultrathin sections of semen sample. (**B1**) A lower-power view of ZIKV particles inside an infected cell, with the characteristic of an immature sperm cell. (**B2**) Viral particles in a magnified view of the same cell in (**B1**). (**B3**) C6/36 cell infected with a semen sample from patient ZIKV17 with a cluster of dense virions located in the cytoplasm (red arrow).

Both males (ZIKV17 and ZIKV 19) had the virus detected in semen and urine and only one (ZIKV17) had it detected in saliva from day 25 (5.2 × 10^6^ ge/mL) through day 32 (4.2 × 10^5^ ge/mL). This patient also showed high viruria at day 6 (2.5 × 10^7^ ge/mL) which waned by day 32 and persistent viral shedding in semen, which was detected from day 18 (6.5 × 10^8^ ge/mL) through day 158 (15 ge/mL) after symptoms onset (Figure 2B). Similarly to patient ZIKV17, prolonged virusemia was detected as early as day 19 (3.13 × 10^5^ RNA ge/mL) through day 82 (1.33 × 10^2^ RNA ge/mL) in the other male patient (ZIKV19) (Figure 2C). The presence of virus in semen has been directly demonstrated by IFA and TEM, where it was verified in both spermatozoids and immature sperm cells (Figure 3, and Figures S1 and S2). All four patients remained negative for DENV and CHIKV RNA detection throughout the study period.

**Figure 4:**
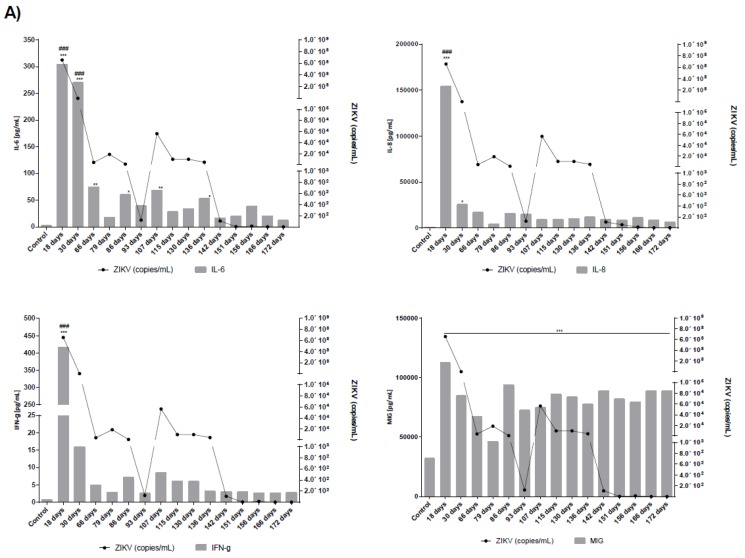

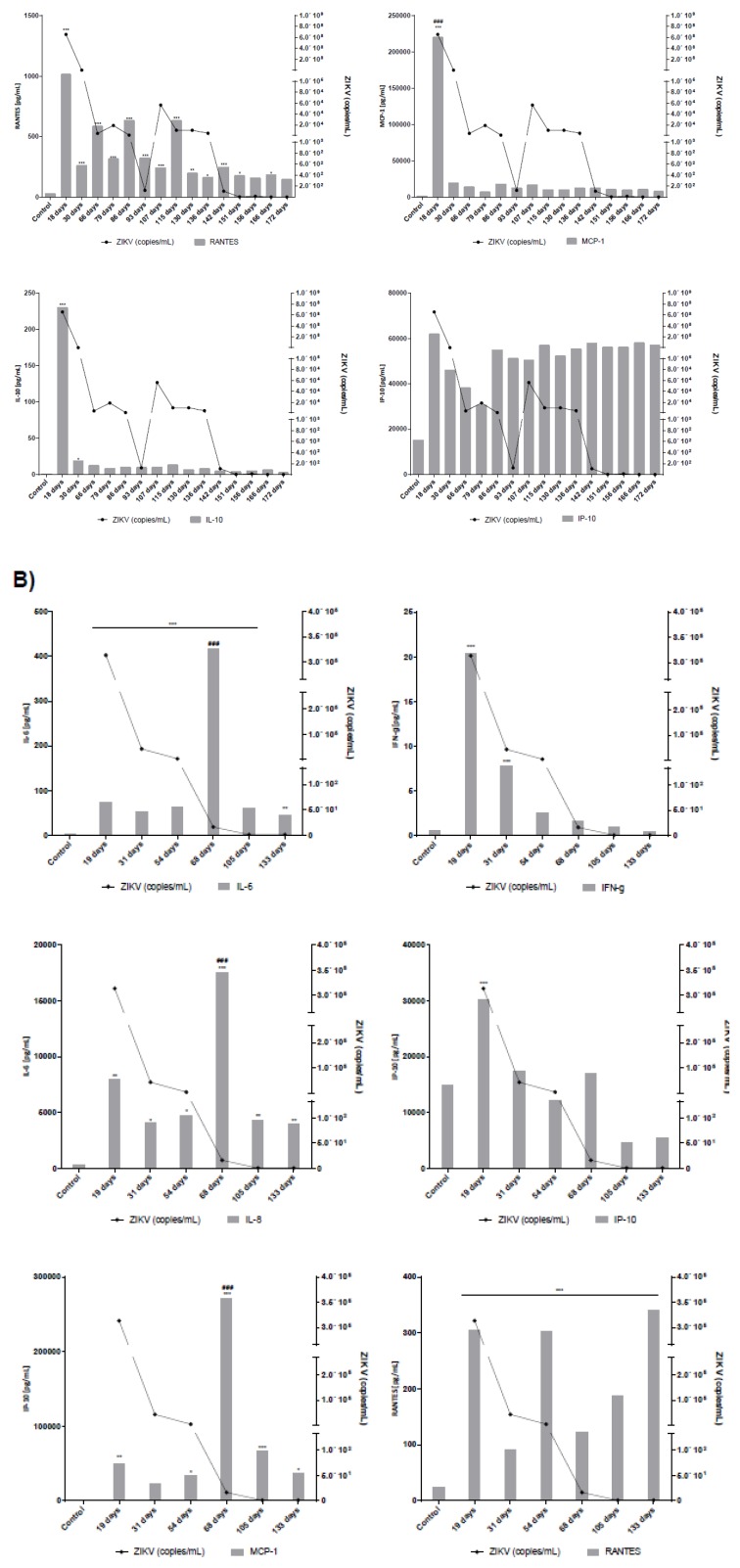
Concentration of cytokines, chemokines and RNA viral load determined on semen of patients ZIKV17 (**A**) and ZIKV19 (**B**). The levels of the following cytokines and chemokines were measured in blood and seminal plasma—IL-2, IL-4, IL-6, CXCL8 (IL-8), IL-10, IL-17, IFN-γ, TNF–α, CCL2 (MCP-1), CCL5 (RANTES), CXCL9 (MIG), CXCL10 (IP-10). The results are representative of two distinct experiments performed in duplicate. Values of *p* less them 0.05 were considered statistically significant (* *p* < 0.05; ** *p* < 0.001; *** *p* < 0.0001—concentration of cytokines/chemokines in ZIKV patients versus control (semen from Zika—uninfected individual), ### *p* < 0.0001 correlation of concentration of cytokine/chemokines in different days after symptoms onset). IL-2, IL-4, IL17A and TNF–α were not detected in the seminal plasma of both patients. No cytokines or chemokines were detected in serum of both patients—serum results were below the limit of detection of the kit (20 pg/mL for cytokines and 10 pg/mL for chemokines).

Replication competent ZIKV particles were successfully isolated from saliva (day 25), urine (days 18, and 25), and semen (days 18, 25, 32, 53, and 117) samples of patient ZIKV17 and from semen samples (days 19, 26 and 40) of patient ZIKV19 (Figure 2B,D). The viral isolation was confirmed by qRT-PCR, IFA and TEM (Figure 3A,B). Although cytokines and chemokines have not been detected in serum by the methodology used, seminal fluids of patients ZIKV17 and ZIKV19 showed enhanced levels of some of them when compared to normal control (semen from a ZIKV-uninfected individual). Patient ZIKV17 showed concentrations of IP-10, MIG, IL-6, IL-10, INF-γ, IL-8, MCP-1and RANTES higher than control, with the highest values found at the beginning of the infection (*p* < 0.05), highlighting a local persistent inflammation in the MRS. Statistical correlation (Pearson’s r) between the Zika load and the level of all these chemokines and cytokines were verified, except for IP-10 (Figure 4A and Supplementary Figure S3A). For patient ZIKV19 the same cytokines/chemokines were higher than control, except by IL-10 that remained undetectable. But a statistical correlation was verified only for IFN-γ and IP-10. In addition, an increased concentration of IL-6, IP-10, MCP-1, IL-8 and RANTES in the seminal plasma sample of this patient was also detected after 60 days of the symptoms onset, which was coincident with the period immediately before the viral clearance (Figure 4B and Supplementary Figure S3B). More, IL-2, IL-4, IL17A and TNF–α were not detected in the seminal plasma of both patients.

### 3.2. ZIKV Evolution during Prolonged Infection 

**Figure 5:**
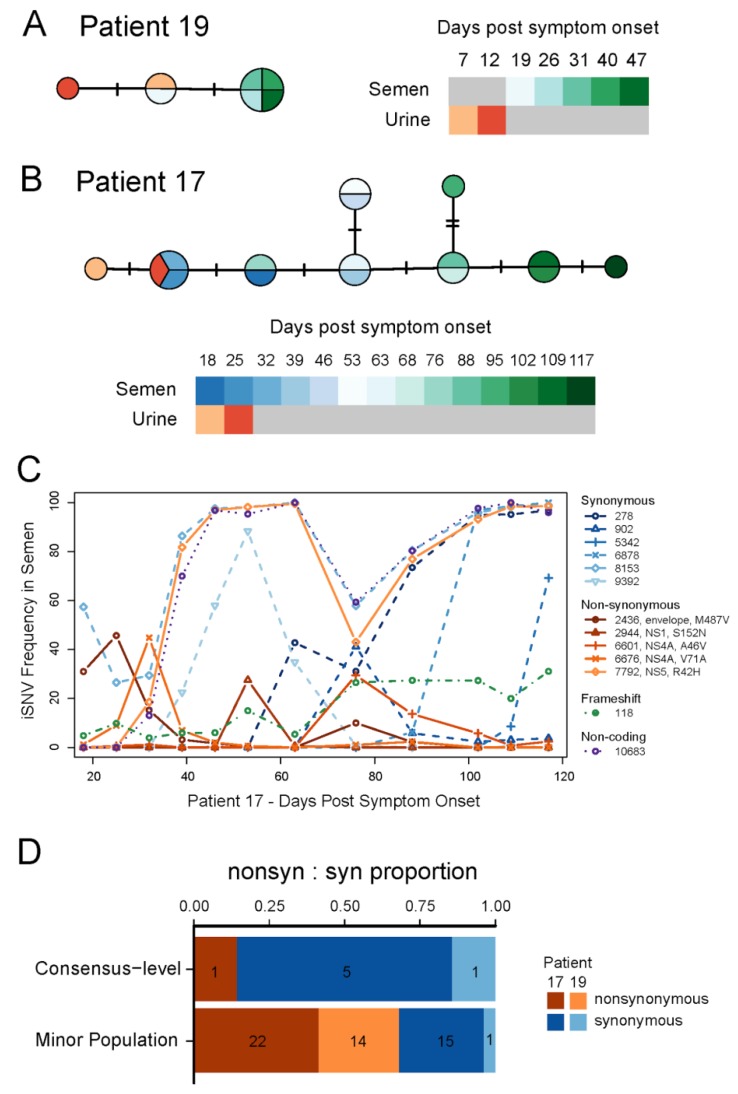
Evolution of ZIKV populations throughout the infection course. Median-joining haplotype networks constructed from full genome alignments of the consensus sequences from patients ZIKV19 (**A**) and ZIKV17 (**B**). Colors indicate sample type and collection date relative to symptoms onset. Each dash represents a single nucleotide substitution differentiating consensus sequences from different samples. (**C**) Intra host single nucleotide variant (iSNV) frequencies over time in semen samples collected from patient ZIKV17. The legend indicates the nucleotide position of each iSNV relative to KX197192.1 (GenBank) and for non-synonymous changes, the affected protein and amino acid change. Only positions with a minimum frequency ≥25% in at least one sample are shown. See Table S3 for details about these mutations and others present at lower frequencies. (**D**) Proportion of nonsynonymous and synonymous ZIKV iSNVs observed in semen samples from patients ZIKV17 and ZIKV19. The relative counts of nonsynonymous and synonymous variants observed above (consensus-level) and below 50% frequency (minor population) in at least one sample were significantly different (Fisher’s exact test *p*-value = 0.01).

The isolation of replicative ZIKV particles from different body fluids, mainly seminal fluids, in the same individuals, particularly the two male patients (ZIKV17 and ZIKV19), allowed us to evaluate the extent of genetic variations observed in prolonged persistence of ZIKV in human hosts. With ZIKV17, we obtained near complete genome sequences directly from fourteen sequentially collected semen samples and two urine samples. For patient ZIKV19, five semen and two urine samples yielded near complete genome sequences (Table S2). We also assembled a near complete ZIKV genome from one urine sample of one female patient (ZIKV18, day 15). Phylogenetic inference indicated that all three patients were infected with viruses closely related to those previously circulating in Brazil (Figure S4). As expected from individual prolonged infections, all of the ZIKV genomes obtained from each patient formed well-supported monophyletic clades (Figure S3). Within both patients, we observed ZIKV genetic changes over time both in consensus sequences (Figure 5A,B) and intra-host single nucleotide variant (iSNV) frequencies (Figure 5C,D, Table S3). This is consistent with active viral replication during prolonged ZIKV infections. With the exception of a few low frequency insertions/deletions associated with homopolymer repeats (Table S3), patterns of genetic variation (iSNVs and consensus-level changes) were distinct between urine and semen samples from the same patient (Figure 5; Table S3), consistent with independent compartmentalization of ZIKV populations in the reproductive and urinary systems.

Using time-structured phylogenies, we estimated the rate of evolution during the prolonged infection of the MRS in ZIKV17. Our estimates were highly consistent across multiple models and were indistinguishable from published rates for the entire ZIKV outbreak in the Americas (Figure S3 and Table S4). ZIKV evolution within the MRS was dominated by synonymous substitutions, consistent with strong purifying selection across most of the ZIKV genome. We observed one nonsynonymous and six distinct synonymous substitutions in the MRS of ZIKV17 (dN/dS via robust counting = 0.06) and only a single synonymous substitution in ZIKV19. We also observed a significant difference between the ratio of nonsynonymous:synonymous variants present at different frequencies in samples with a high depth of coverage. Synonymous changes were most prevalent among variants that reached high frequencies (≥50%) during the course of infection, while nonsynonymous changes were more common in variants that were maintained at low frequencies (Figure 5D; Fisher’s exact test *p*-value = 0.01). This pattern is consistent with incomplete purifying selection acting on low frequency variants [25].

**Figure 6:**
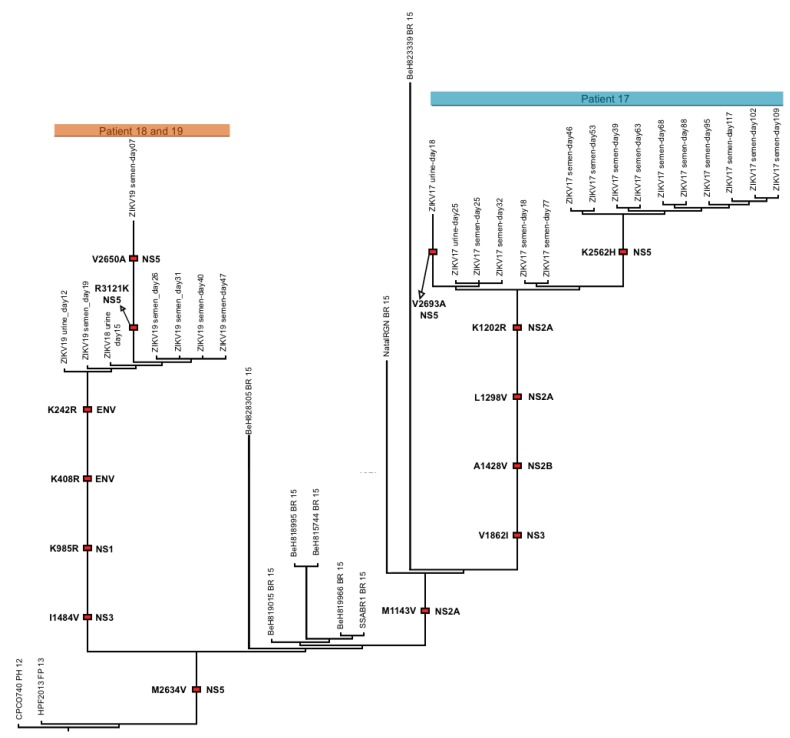
Dendogram showing the most parsimonious unique amino acid changes with high consistency index (CI=1) (black framed red boxes). Reconstructions were made using a set of ZIKV polyproteins from African and Asian lineage viruses. Branch lengths are shown proportional to the number of most parsimonious reconstructions (MPR) of amino acid changes. Amino acid changes that define patient clades are shown as well as the viral proteins affected. Each patient clade (which had 100% support in ML tree shown in Figure S3) was supported by four synapomorphic changes. For both patients ZIKV19 and ZIKV17 changes were observed in the NS5 protein. Although we only show the results for selection detection methods for the three patients, elevated rates of non-synonymous changes were detected for all of the codons containing unique amino acid changes shown. The multiple EM for motif elicitation (MEME) algorithm detected significant positive selection (*p*-value = 0.03) acting on the codons containing the two NS5 changes observed during infection of patient ZIKV17. All MPRs were detected with FUBAR with a Bayes factor >3 and had elevated *dN*. Sites detected by 2-rates FEL had nonsynonymous changes in the absence of detectable synonymous changes. Significant negative, purifying selection was detected by all methods used on several sites of the polyprotein.

Despite the overall signal of purifying selection, positive diversifying selection may have affected some regions of the genome. We utilized several phylogenetic-based methods to look for signatures of positive selection at the codon-level. The reconstructed most parsimonious amino acid changes along the ZIKV ML tree topology (Figure 6, Table S5) indicated four synapomorphic changes (K242R, K408R, K985R and I1484V) defining the lineage infecting both patients ZIKV18 and ZIKV19. In addition, the virus in the urine sample of patient ZIKV19 had two additional unique changes in the NS5 peptide (V2650A and R3121K); however, these changes were not observed after the removal of duplicate sequencing reads. Patient ZIKV17 had four defining synapomorphic changes (K1202R, L1298V, A1428V and V1862I). In the urine samples, only one substitution in the NS5 (E2693G) was identified (present in one sample). Semen samples from patient ZIKV17 had a change in the NS5 (R2562H) that was present in genomes reconstructed from eight time points (39, 53, 63, 68, 88, 95, 102 and 109 days after onset symptoms). Interestingly, the consensus genome showed the initial state (2562R) at day 76, which suggests that distinct haplotypes were circulating at varying frequencies over time. Substitutions observed in patients ZIKV17 and ZIKV19 did not alter the conformation of the proteins as measured by homology modeling and structural alignments.

## 4. Discussion

The aim of the present study was to follow the kinetics of ZIKV persistence and secretion in different host compartments and body fluids. The study included four (two males and two females) ZIKV-infected symptomatic adults who were followed for up to 298 days. Our initial conclusions supported previous evidence of prolonged ZIKV shedding in the semen of several different mammalian species, including humans [13,16,17,26,27] and confirmed the importance of semen analyses as a diagnostic tool for males with ZIKV disease. The study also confirmed the presence of ZIKV particles inside spermatic cells and seminal fluids as late as 117 days post the initial onset of symptoms. In contrast to previous reports based on the detection of RNA material, our results confirmed that complete infectious viral particles can be detected in the semen up to 117 days after the onset of symptoms.

On the other hand, the early detection of anti-ZIKV IgG and IgM antibody responses and the rapid clearance of ZIKV from serum strongly suggest that the immune system can easily handle the systemic phase of the infection. The detection of proinflammatory cytokine responses and the presence of infectious virus in seminal fluids long after viral clearance from serum indicate that the local immune system does not efficiently control ZIKV replication in the MRS. Similarly to other flaviviruses, ZIKV may promote some sort of local immunosuppression allowing viral replication despite activation of systemic immune responses. In cases of DENV infection, the virus is capable of antagonizing the innate host responses using multiple strategies including degradation of key proteins, such as STAT2, 2’-O, methylation of adenosine in the viral genome and interfering with RNAi mechanisms [28,29,30]. It remains to be seen if ZIKV proteins are capable of exerting local negative regulatory effects on the host’s immune system, which may contribute to viral persistence. Besides, the alteration in the genital cytokine milieu could play an important role in replication and transmission of the virus as has been seen for other viruses, such as HIV-1 [31], which could considerably increase the risk of ZIKV sexual spread and potentially play a role on the viral spread. In addition, despite, experimental evidence in mice that persistent ZIKV infections can cause infertility in males [17], patient ZIKV17 was able to inseminate his wife approximately two months after virus clearance and the couple had a healthy baby.

The availability of ZIKV-containing semen and urine samples of two patients allowed us to study the genetic evolution of the virus during prolonged replication in the MRS. For the first time, we demonstrated a high level of evolutionary constraint on ZIKV during the prolonged MRS infections. High levels of constraint, as evidenced by low long-term rates of amino acid substitution, have commonly been observed for arthropod-borne viruses like ZIKV [32]. However, the primary source of this constraint is generally thought to be the continual alternation of these viruses between vertebrate and invertebrate hosts, each of which exerts unique selective pressures. By examining ZIKV genetic diversity over time in the semen of patients with prolonged MRS infections, we obtained a rare glimpse into the selective pressures exerted by a single host species. Although a large number of nonsynonymous variants were detected within both patients, few of these mutations ever reached high frequencies (Figure 5). In fact, our estimate of dN/dS from the semen samples of ZIKV17 is very similar to recent long-term dN/dS estimates for ZIKV, which average across the selective pressures from both vertebrate and invertebrate hosts [7]. Our findings, therefore, indicate that the strong evolutionary constraint observed for ZIKV and other arthropod-borne viruses may not simply represent the long term effect of alternating between hosts, but is likely also related to the pressures experienced within individual host species.

## Supplementary Materials

The following are available online http://www.mdpi.com/1999-4915/10/11/615/s1: Figure S1: ZIKV characterization by IFI in semen samples, Figure S2: ZIKV detection in semen samples from patient ZIKV17; Figure S3: Concentration of cytokines, chemokines, RNA viral load and laboratorial follow-up of patients ZIKV17 and ZIKV19, Figure S4: Significantly higher proportion of nonsynonymous mutations in low frequency variants; Table S1: qRT-PCR Ct obtained from body fluid samples; Table S2: Zika virus genomes produced in this study; Table S3: Intra-host variant summary; Table S4: Substitution rate estimates for ZIKV during prolonged infection of male reproductive system; Table S5: Probes used for Zika virus targeted enrichment; Table S6: Algorithms for genetic evolution; Table S7: GenBank accessions for the consensus genomes.
