# Supplementary material for: Persistence and Intra-Host Genetic Evolution of Zika Virus Infection in Symptomatic Adults: A Special View in the Male Reproductive System
Source: Viruses. 2018 Nov 7;10(11):615. doi: 10.3390/v10110615 (PMC6267439; doi:10.3390/v10110615)
Supplement: Supplementary file 1 [file viruses-10-00615-s001.pdf]

SUPPLEMENTARY APPENDIX

Title

Persistence and Intra-Host Genetic Evolution of Zika Virus Infection in Symptomatic Adults: a Special View in the Male Reproductive System

Table of content

ADDITIONAL METHODS………………………………………………………… 1

Real-time PCR………………………..……………………………………… 1

Virus Isolation in semen patient ZIKV19……....………………………… 1

Virus detection in semen patient ZIKV17..……..………………………… 2

ZIKV Enzyme-linked immunosorbent assay (ELISA)……………………… 3

Genome sequence…………………………..….….………………………. 4

Genome assembly and analysis…………………………………………….. 4

ZIKV evolution during prolonged infection ………………………………… 5

Supplementary FIGURE ……………..……….....………………………... 9

Figure S1- ZIKV characterization by IFI in semen samples………………… 7

Figure S2. ZIKV detection in semen samples from patient ZIKV17 and isolates from cell culture …………………………………………………….... 8

Figure S3 - Concentration of cytokines, chemokines, RNA viral load and laboratorial follow-up of patients ZIKV17 and ZIKV19…………………….. 9

Figure S4- Significantly higher proportion of nonsynonymous mutations in low frequency variants…………………………………………………….... 11

Supplementary TABLE …………………………………………………. 12

Table S1- qRT-PCR Ct obtained from body fluid samples……………….. 12

Table S2- Zika virus genomes produced in this study …………………… 13

Table S3- Intra host variant summary …………………………..…….. 12

Table S4- Substitution rate estimates for ZIKV during prolonged infection of the male reproductive system ………..……..………………… 12

Table S5a and b- Probes used for Zika virus targeted enrichment …..… 13

Table S6- Algorithms for genetic evolution ……………………………….. 13

Table S7 - GenBank accessions for the consensus genomes ………….. 14

Supplementary REFERENCES…………………………………………….. 14

1. METHODS

Quantitative reverse-transcription PCR (qRT-PCR). RNA sample was extracted from 300µl samples of saliva, 400µl of urine or serum and 100 µl of semen, with NUCLISENS ® easyMag ® (BioMerieux) on the platform "off board"- pre lysed sample. Briefly, lysis buffer was added in a proportion of 3:1 to sample. The mixture was incubated for 10 minutes at room temperature and after this time we added 30 µl of magnetic silica and take the Easymag machine. The RNA was eluted in 40 µl of elution buffer and stored at-80 ^{0}C until the time of use. All extracts were tested for the presence of human RNase P gene by RT-PCR to confirm sample quality. The PCR reaction was carried out with RNA from each sample with a set of primers and probes with FAM as dye reporter for the probe. Primers/probes for Zika virus (ZIKV), Dengue virus (DENV) and Chikungunya (CHIKV) were previously described [10, 12, 18]. All assays were performed using the AgPath-IDTM One-Step qRT-PCR reagents (Applied Biosystems). Succinctly, we used 5 µl of the RNA extracted in 1µl of the mix from primers/probe [10pM/µl] and 19 µl of the reagent mix from AgPath-IDTM One-Step RT-PCR kit following manufacturer's instructions. To ensure RNA integrity and sample quality, all extracts were also tested for human RNase P (RNP) gene by qRT-PCR. All extracts showed robust RNase P values ranging from cycle threshold (*Ct)* values, which ranged from 16.3 to *Ct* 33.5 (see Supplementary Appendix Table S1 for details) demonstrating the integrity of the material collected. Assays for DENV [18] and CHIKV[12] were performed as controls for co-infection, as they have been reported in Brazil during the ZIKV outbreak.

Virus isolation in cell culture. Presence of viral RNA in a biological specimen is not equivalent to demonstration of infectivity. Thus, we attempted virus isolation of ZIKV from RNA positive semen, saliva and urine samples collected from both male patients to confirm presence of infectious virus. For virus isolation, *Aedes albopictus* mosquito cells (C6/36) were inoculated with 500 µl of saliva, urine or semen. After 8-12 days of incubation, cells were collected and tested for ZIKV

presence by qRT-PCR. All samples were passaged at least 3 times in cell culture before being considered negative. Briefly, C6/36 cell culture was maintained using Leibovitz's L-15 medium (L15) supplemented with 10% fetal bovine serum (FBS) (Gibco©), 1% non-essential amino acids (Gibco©), 1% sodium piruvate (Gibco©), 1% penicillin/streptomycin (Gibco©), 0.05% of Fungizone® Amphotericin B (Gibco©) and kept at 27°C in the absence of CO_2. After reaching approximately 70% of confluence in the monolayer, we prepared a 1:1 mix with L15 medium and 500 µl of urine or semen and inoculated it into C6/36 cells. We allowed one hour for adsorption, with gentle shaking every 10 minutes to ensure homogeneous adsorption of the viruses. At the end of the adsorption period, 5mL of the complete L-15 culture media were added. After 8-12 days of incubation, cells were collected and tested for ZIKV presence by qRT-PCR. All the samples were passaged at least 3 times in cell culture and ZIKV presence were confirmed by qRT-PCR.

Virus Detection in Semen (IFA and TEM). Serum of patients ZIKV17 and ZIKV19 (positive for anti-ZIKV IgG at day 14 and 19 after symptom onset, respectively) and an anti-flavivirus monoclonal antibody 4G2 were used as primary antibody, an anti-mouse IgG-FITC (Sigma-Aldrich®) and an anti-human IgG-FITC (Sigma-Aldrich®) were used as secondary antibodies. Following incubation and a 10 min wash in PBS, slides were mounted in glycerol and examined for the presence of fluorescently labeled cells using a fluorescence microscope (FITC/Olympus BX41) or confocal microscope. Samples were also stained with DAPI for nucleus. Semen samples from a healthy ZIKV non-infected control donor were used as negative control. For TEM samples were fixed in a solution of 2.0% glutaraldehyde, 2.0% paraformaldehyde, 2.5mM calcium chloride, 0.1M sodium cacodylate, pH7.3 for at least 72h at 4ºC followed by centrifugation at 500 g for 15 minutes. Samples were then rinsed with the same buffer 0.1 M, pH 7.2, post-fixed for 2 h at room temperature in 1.0% osmium tetroxide (Electron Microscopy Sciences, USA) in the same buffer, dehydrated through increasing concentrations of ethanol up to 100%, and embedded in Spur resin (Electron Microscopy Sciences, USA). Sections (0.5µm thick) of semen samples were stained with 1.0% toluidine blue in 1.0% aqueous sodium borate for light microscopic examination. Thin sections were stained with uranyl acetate and lead citrate and observed with a JEOL 1010 transmission electron

microscope.

ZIKV Enzyme-linked immunosorbent assay (ELISA). As a confirmatory test for ZIKV, we utilized an “in house” ELISA assay using ZIKVΔNS1 recombinant protein for IgG detection. Briefly, the polystyrene COSTAR microplates (Corning Inc., New York, USA) were coated with 200 ng of the recombinant protein dilute in carbonate-bicarbonate buffer pH 9.6, incubated overnight at 4°C and, then, blocked with a blocking solution for 2 h at room temperature. The plates were washed three times with PBS-TWEEN 0.05% (PBS-T) and followed by addition of the serum samples diluted 1:100 in blocking solution, and subsequently incubated at 37ºC for 60 min. After a new washing cycle, the anti-human IgG peroxidase-conjugated (Aldrich Sigma, USA) was added to wells and further incubated for 60min. After a final washing step TMB substrate was added and the reaction was stopped after 15 min with the addition of 50µL of H_2SO_4 at 0.1M. The optical density of the reaction was measured at 450 nm in a microplate reader (Labsystems Multiscan, ThermoScientific, USA). ZIKV-specific IgM antibodies were detected using capture ELISA with a specific viral antigen for ZIKV [(14)].

Genome sequencing. All samples were sequenced at the US Army Medical Research Institute of Infectious Diseases. Sequencing libraries were prepared using the TruSeq RNA Access Library Prep kit (Illumina, Menlo Park, CA) with custom ZIKV probes. The set included 866 unique probes each of which was 80 nt in length (Supplemmentary Table 5B - TS5). The probes were designed to cover the entire ZIKV genome and to encompass the genetic diversity present on GenBank on January 14, 2016. In total, 26 ZIKV sequences were used during probe design (TS5). Extracted RNA was fragmented at 94˚C for 0-60 seconds and each sample was enriched separately using a quarter of the reagents specified in the manufacturer’s protocol. Samples were bar coded, pooled and sequenced using the MiSeq Reagent kit v3 (Illumina, Menlo Park, CA) on an Illumina MiSeq with a minimum of 2x151 bp reads. Dual indexing, with no overlapping indices, was used to prevent bleed through between samples. These two approaches resulted in nearly identical sequences. However, for several lower coverage samples, contiguous assemblies could only be constructed with the reference-based approach. Consensus genomes were compared using median-joining haplotype networks (PopART v1.7.2) and an

approximate maximum-likelihood phylogenetic reconstruction (FastTree v2.1.5). BEAST v1.8.3 [(5)] was used to estimate dN/dS and the rate of ZIKV evolution within the male reproductive system (MRS), HyPhy v2.3 was used to identify codons with evidence for positive, diversifying selection, and for samples with >50x average coverage, we examined intra host genetic variation using FreeBayes v1.0.2 [(7)].

Genome assembly and analysis. Consensus genomes based on alignments to a reference were generated as previously described by Blackley et al. (2016) with KU365778.1 as a reference [(2)]. Additionally, genomes were assembled de novo using the CLC Genomics Workbench. Prior to de novo assembly, Illumina adaptors were clipped and reads were quality trimmed and filtered using Trim Galore!. Haplotype networks were generated with PopART version 1.7.2 (http://popart.otago.ac.nz) using the median-joining reconstruction method. FreeBayes v1.0.2 was used to detect intra host single nucleotide variants (iSNVs) [(7)]. For iSNV detection, we mapped reads to custom reference genomes for each patient. For patient ZIKV17, the reference used was equivalent to the consensus sequences assembled from the patient's urine sample from day 25 and semen samples from days 25 and 32. For patient ZIKV19, the reference was equivalent to the consensus sequences assembled from the patient's urine sample from day 7 and semen sample from day 19. We only used reads with mapping quality ≥30 and positions with base quality ≥30, and an iSNV was only considered if the alternate allele was represented by ≥5 reads and present at a frequency ≥3% in at least one sample from the patient.

Genomes for each patient time point were aligned with 38 ZIKV genomes, representing the known ZIKV lineages that were circulating in Brazil, using MAFFT available in Geneious v. 9.0.5, which was further refined by hand using Geneious alignment editor. An approximate maximum likelihood (ML) phylogenetic tree for the complete open reading frame (ORF) of all ZIKV genomes was generated using FastTree v2.1.5 with a SH-like local supports. The ML topology was used to

reconstruct and map the most parsimonious amino acid changes with MacClade v4.07. The ML tree was also used to look for evidence of positive diversifying selection at the codon-level in the ZIKV ORF using the SLAC, 2-rates FEL, MEME and FUBAR (TS6) algorithms implemented in HyPhy v2.3 [16]. Ancestral states that define each clade (i.e., synapomorphic changes) were then further investigated by replacing them onto structural models for ZIKV peptides built with YASARA energy minimization and structural alignments were carried and variations in structure due to the selected amino acid changes were recorded. The structures were then evaluated for linear and discontinuous epitopes using the online servers IEDB and BePRO [17].

BEAST v1.8.3 [5] was used to estimate dN/dS and the rate of ZIKV evolution within the male reproductive system (MRS). For these analyses, we utilized the ORF sequences for the 14 genomes we assembled from semen samples from patient ZIKV17. ZIKV17 experienced the most prolonged MRS infection in this study. We partitioned sites into codon positions 1, 2 and 3 and used independent HKY substitution models. To estimate substitution rate, we included $\Gamma^{(4)}$-distributed rate heterogeneity and tested multiple combinations of molecular clocks (strict and relaxed with lognormally distributed rate categories) and coalescent priors (constant size, Bayesian Skygrid with 10 parameters and Bayesian Skyline with 10 groups).[3-5, 8, 9] We compared models using marginal likelihood comparison with path sampling and stepping-stone estimation approaches [1]. For estimating dN/dS, we used the renaissance counting [11, 13] option with the simplest molecular clock and coalescent prior combination (strict and constant size). All of these Bayesian analyses were run for 100 million Markov chain Monte Carlo steps, sampling parameters and trees every 10,000 generations.

ZIKV EVOLUTION DURING PROLONGED INFECTION. For patient ZIKV17, we obtained near complete genome sequences from fourteen sequentially collected semen samples and two urine samples. For patient ZIKV19 five semen and two urine samples yielded near complete genomes (Table S2). With the exception of a few low frequency insertions/deletions associated with homopolymer repeats (Table S3), patterns of genetic variation (iSNVs and consensus-level changes) were distinct between urine and semen samples from the same patient (Figure 6; Table S3),

consistent with independent compartmentalization of ZIKV populations in the reproductive and urinary systems. Using time-structured phylogenies, we estimated the rate of evolution during the prolonged infection of the MRS in ZIKV17. Our estimates were highly consistent across multiple models and were indistinguishable from published rates for the entire ZIKV outbreak in the Americas (Figure S3, Table S4). ZIKV evolution within the MRS was dominated by synonymous substitutions, consistent with strong purifying selection across most of the ZIKV genome. We observed one nonsynonymous and six distinct synonymous substitutions in the MRS of ZIKV17 (dN/dS via robust counting = 0.06) and only a single synonymous substitution in ZIKV19. We also observed a significant difference between the ratio of nonsynonymous:synonymous variants present at different frequencies in samples with a high depth of coverage. Synonymous changes were most prevalent among variants that reached high frequencies (≥50%) during the course of infection, while nonsynonymous changes were more common in variants that were maintained at low frequencies (Figure 5D; Fisher's exact test p-value = 0.01). This pattern is consistent with incomplete purifying selection acting on low frequency variants[(15)]. In addition, the virus in the urine sample of patient ZIKV19 had two additional unique changes in the NS5 peptide (V2650A and R3121K); however, these changes were not observed after the removal of duplicate sequencing reads. Patient ZIKV17 had 4 defining synapomorphic changes (K1202R, L1298V, A1428V and V1862I). In the urine samples, only one substitution in the NS5 (E2693G) was identified (present in one sample). Semen samples from patient ZIKV17 had a change in the NS5 (R2562H) that was present in genomes reconstructed from eight time points (39, 53, 63, 68, 88, 95, 102 and 109 days after onset symptoms). Interestingly, the consensus genome showed the initial state (2562R) at day 76, which suggests that distinct haplotypes were circulating at varying frequencies over time. Substitutions observed in patients ZIKV17 and ZIKV19 did not alter the conformation of the proteins as measured by homology modeling and structural alignments (data not shown).

2- SUPPLEMENTARY FIGURE

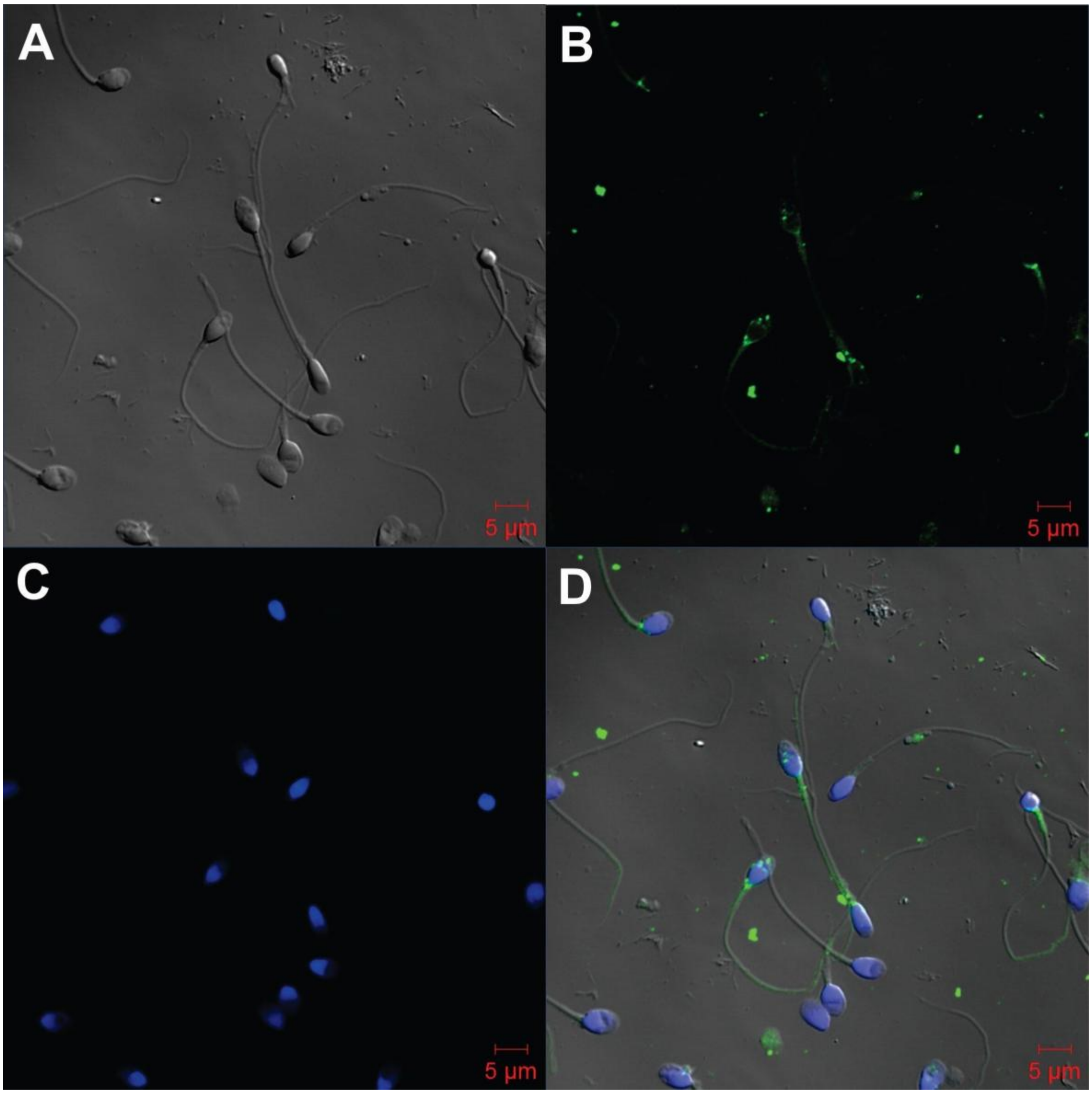

Figure S1. Confocal microscopy for ZIKV detection using immunofluorescence in semen samples of patient 19. (**A**) Sample observed in transmitted light; (**B**) FITC demonstrating ZIKV presence located into cytoplasm and flagella of spermatozoa from patient (day 40); (**C**) The sample observed in DAPI channel; (**D**) images overlap.

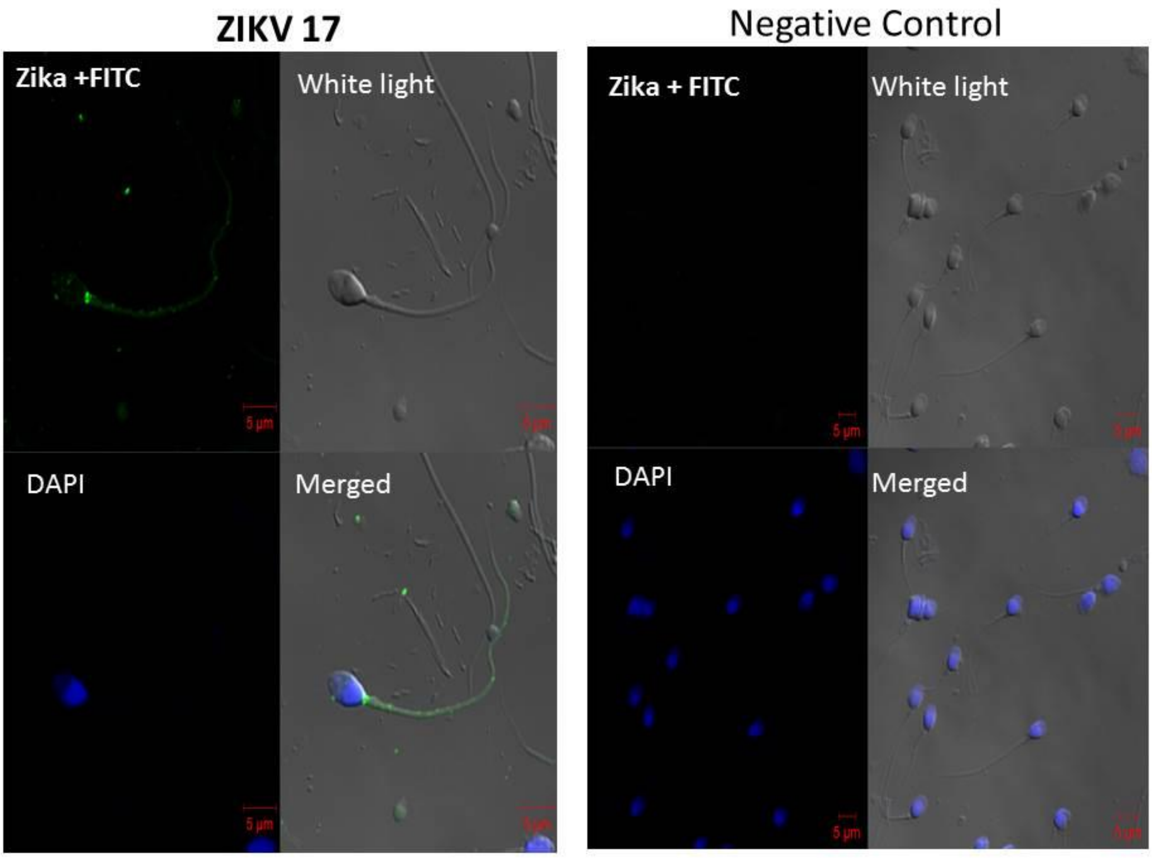

Figure S2. ZIKV detection in semen samples from patient ZIKV17. Samples of semen were stained for detection of ZIKV by IFI. The sample observed in DAPI channel is represented in blue and ZIKV appears stained with FITC (green), demonstrating ZIKV presence located into cytoplasm and flagella of spermatozoa from patient ZIKV17.

A)

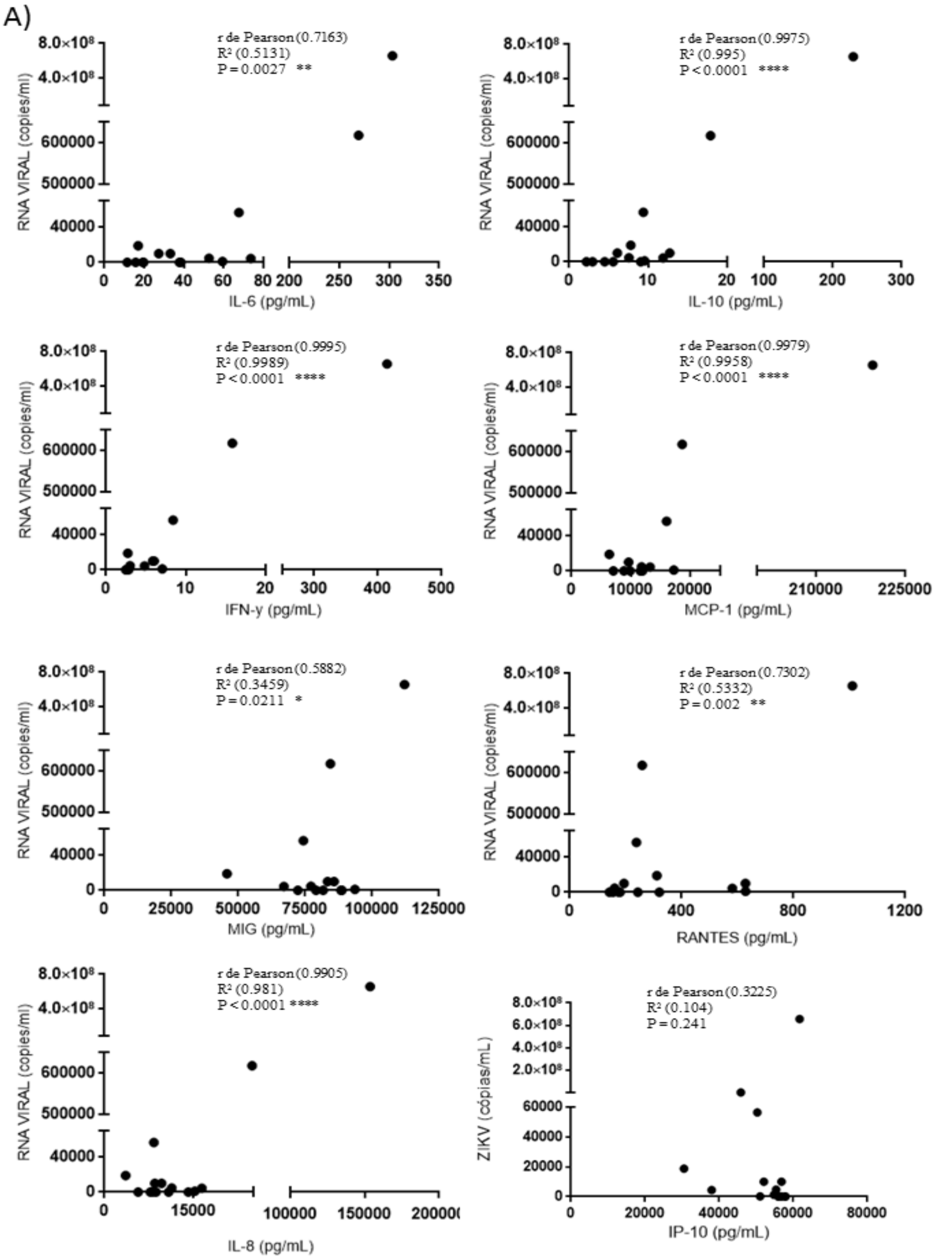
r de Pearson (0.7163)
R² (0.5131)
P = 0.0027 **
IL-6 (pg/mL)
r de Pearson (0.9975)
R² (0.995)
P < 0.0001 ****
IL-10 (pg/mL)
r de Pearson (0.9995)
R² (0.9989)
P < 0.0001 ****
IFN-y (pg/mL)
r de Pearson (0.9979)
R² (0.9958)
P < 0.0001 ****
MCP-1 (pg/mL)
r de Pearson (0.5882)
R² (0.3459)
P = 0.0211 *
MIG (pg/mL)
r de Pearson (0.7302)
R² (0.5332)
P = 0.002 **
RANTES (pg/mL)
r de Pearson (0.9905)
R² (0.981)
P < 0.0001 ****
IL-8 (pg/mL)
r de Pearson (0.3225)
R² (0.104)
P = 0.241
IP-10 (pg/mL)
RNA VIRAL (copies/ml)
ZIKV (cópias/mL)

B)

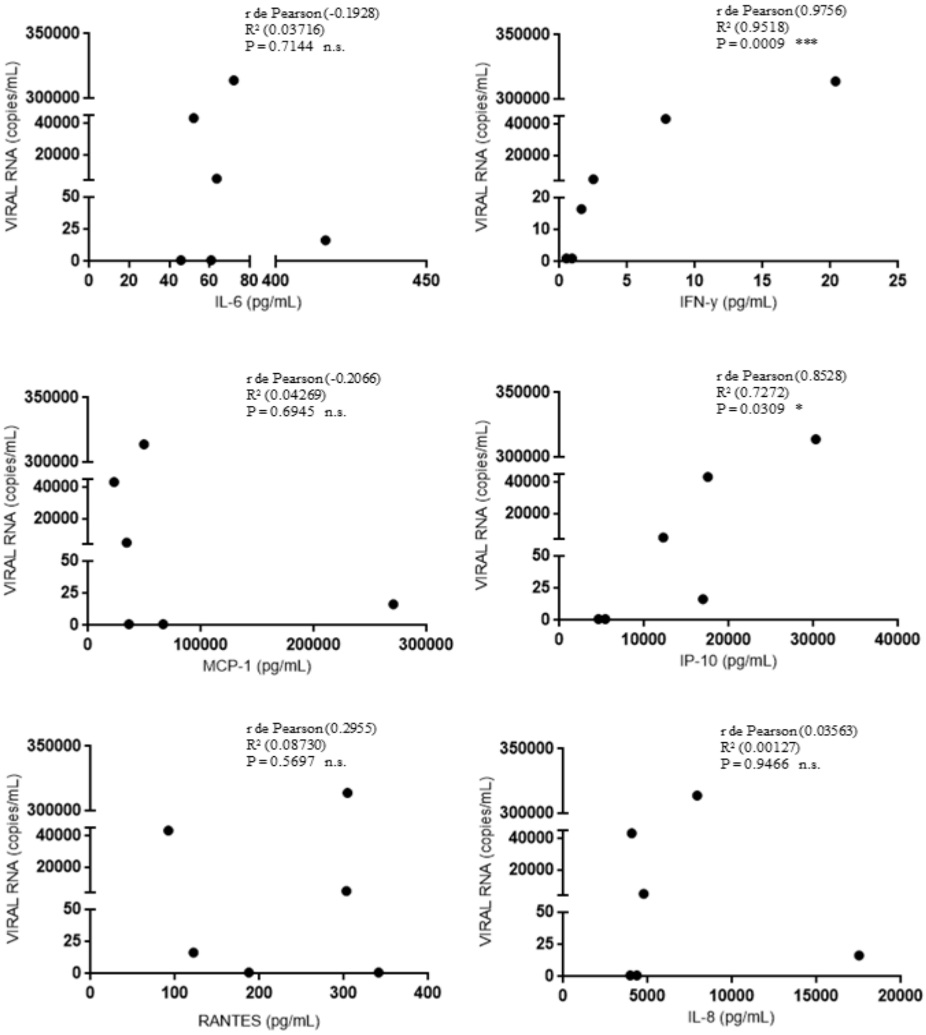

Figure S3. Correlation between Zika virus loads and concentration of cytokines and chemokines**.** Correlation between Zika virus loads in seminal plasma during the clinical and laboratorial follow up of patients ZIKV17 (**A**) and ZIKV19 (**B**) and concentration of cytokines and chemokines. The results are representative of two distinct experiments performed in duplicate. For the correlation analyses, the Pearson (r) correlation coefficient was used and statically significant values of p less than 0.05 were considered. The levels of the following cytokines and chemokines were measured - IL-2, IL-4, IL-6, CXCL8 (IL-8), IL-10, IL-17, IFN-γ, TNF–α, CCL2 (MCP-1), CCL5 (RANTES), CXCL9 (MIG), CXCL10 (IP-10). The cytokines and chemokines from blood of both patients were undetectable. IL-2, IL-4, IL17A and TNF- α were not detected in the seminal plasma of both patients. A) ZIKV17 - A positive correlation between load viral and the level of MIG, IL-6, IL-10, INF- γ, IL-8, MCP-1and RANTES was verified; B) ZIKV19- A positive correlation was verified only between the RNA Zika load and the level of IFN-γ e IP-10.

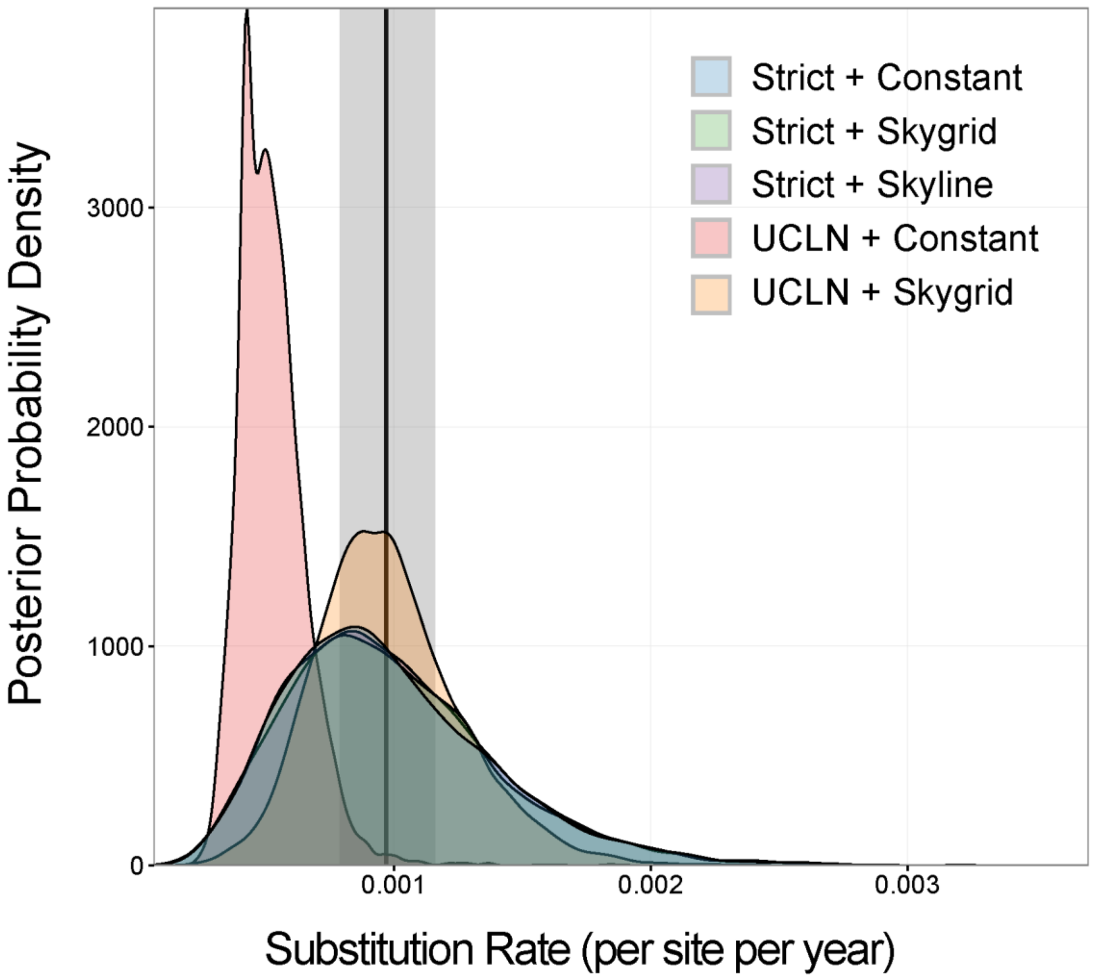

Figure S4. Substitution rate estimates for ZIKV from ZIKV17 semen samples are generally consistent with long-term ZIKV rate estimates. Substitution rate was estimated using several different molecular clock and coalescent model combinations in BEAST v1.8.3 (see TS4). UCLN: uncorrelated relaxed clock with lognormal distribution. The vertical black line represents the mean long-term ZIKV rate estimate from Faria et al. 2017[6] with the 95% HPD shaded in grey.

3- SUPPLEMENTARY TABLE

TS1- qRT-PCR Ct values obtained from body fluid samples.

Samples	Low Ct[1]		Ct mean[2]		High Ct	
	RNP[3]	ZIKV[4]	RNP	ZIKV	RNP	ZIKV
01URI[5]	21.84	33	27.98	35.85	29.14	38.7
01S.O[6]	22.02	35.74	24.27	35.74	26.52	35.74
01Serum	27.80	18	29.59	25.17	31.96	32.34
17URI	17.32	24.73	26.635	25.56	31.55	26.39
17S.O	19.24	0	21.31	0	26.05	0
17Serum	24.43	0	27.47	0	33.57	0
17Semen	17.17	11.46	17.8	28.08	20.24	38.07
18URI	24.19	24.56	29.82	27.195	33.08	29.83
18S.O	22.46	0	24.43	0	28.38	0
18Serum	24.98	0	27.69	0	32.30	0
19URI	18.14	24.83	24.87	29.09	29.7	34.5
19S.O	18.43	0	19.6	0	20.76	0
19Serum	20.2	0	24.11	0	26.14	0
19Semen	16.3	22.03	19.45	26.44	26.47	36.03

[1] Threshold cycle. [2]RNP and ZIKV qRT-PCR Ct mean of all the collected samples were calculated separately for each type of body fluid. [3] Human RNase P. [4]Zika virus. [5] Urine. [6] Oral swab.

TS2- Zika virus genomes produced in this study.

Patient	Sample type	Onset	Collection	Days post onset	# Genome nt covered	# CDS nt covered	% Coverage (genome)	% Coverage (CDS)	Avg Coverage Depth*	# nt with >50x cov	GenBank Accession	ID
17	semen	4/1/16	4/19/16	18	10753	10272	99.49%	100.00%	3410.4	10746	tbd	ZKV17sem_2016-04-19
17	semen	4/1/16	4/26/16	25	10753	10272	99.49%	100.00%	1122.1	10718	tbd	ZKV17Asem_2016-04-26
17	semen	4/1/16	5/3/16	32	10752	10272	99.48%	100.00%	328.7	10708	tbd	ZKV17Bsem_2016-05-03
17	semen	4/1/16	5/10/16	39	10752	10272	99.48%	100.00%	148.6	10599	tbd	ZKV17Csem_2016-05-10
17	semen	4/1/16	5/17/16	46	10752	10272	99.48%	100.00%	412.5	10675	tbd	ZKV17Dsem_2016-05-17
17	semen	4/1/16	5/24/16	53	10752	10272	99.48%	100.00%	148.0	10621	tbd	ZKV17Esem_2016-05-24
17	semen	4/1/16	6/3/16	63	10725	10272	99.23%	100.00%	149.0	10364	tbd	ZKV17Fsem_2016-06-03
17	semen	4/1/16	6/8/16	68	10753	10272	99.49%	100.00%	33.6	922	tbd	ZKV17Gsem_2016-06-08
17	semen	4/1/16	6/16/16	76	10777	10272	99.71%	100.00%	119.8	10666	tbd	ZKV17Hsem_2016-06-16
17	semen	4/1/16	6/28/16	88	10782	10272	99.76%	100.00%	109.0	10648	tbd	ZKV17Jsem_2016-06-28
17	semen	4/1/16	7/5/16	95	10167	9883	94.07%	96.21%	9.2	0	tbd	ZKV17Ksem_2016-07-05
17	semen	4/1/16	7/12/16	102	10784	10272	99.78%	100.00%	118.2	10672	tbd	ZKV17Lsem_2016-07-12
17	semen	4/1/16	7/19/16	109	10694	10272	98.95%	100.00%	148.7	10477	tbd	ZKV17Msem_2016-07-19
17	semen	4/1/16	7/27/16	117	10731	10272	99.29%	100.00%	94.6	10382	tbd	ZKV17Nsem_2016-07-27
17	urine	4/1/16	4/19/16	18	10752	10272	99.48%	100.00%	562.0	10717	tbd	ZKV17uri_2016-04-19
17	urine	4/1/16	4/26/16	25	10752	10272	99.48%	100.00%	173.9	10669	tbd	ZKV17Auri_2016-04-26
18	urine	4/27/16	5/12/16	15	10217	9935	94.53%	96.72%	9.8	0	tbd	ZKV18Buri_2016-05-12
19	semen	4/23/16	5/12/16	19	10767	10272	99.62%	100.00%	3307.7	10753	tbd	ZKV19Bsem_2016-05-12
19	semen	4/23/16	5/19/16	26	10791	10272	99.84%	100.00%	6399.9	10775	tbd	ZKV19Csem_2016-05-19
19	semen	4/23/16	5/24/16	31	10791	10272	99.84%	100.00%	3143.7	10780	tbd	ZKV19Dsem_2016-05-24
19	semen	4/23/16	6/2/16	40	10752	10272	99.48%	100.00%	199.5	10344	tbd	ZKV19Esem_2016-06-02
19	semen	4/23/16	6/9/16	47	10782	10272	99.76%	100.00%	411.2	10708	tbd	ZKV19Fsem_2016-06-09
19	urine	4/23/16	4/30/16	7	10654	10142	98.58%	98.73%	25.3	810	tbd	ZKV19uri_2016-04-30
19	urine	4/23/16	5/5/16	12	10792	10272	99.85%	100.00%	82.3	8724	tbd	ZKV19Auri_2016-05-05

Legend: *Average only includes nt with at least 1x coverage
CDS: Coding sequence; nt:nucleotide; Avg: average

TS3- Intra host variant summary- file:///Users/danielledurigon/Desktop/TSupplementary%20NEJM%20apr17/TS3/ZIKV17.html
file:///Users/danielledurigon/Desktop/TSupplementary%20NEJM%20apr17/TS3/ZIKV19.html

TS4- Substitution rate estimates for Zika virus during prolonged infection of the male reproductive system in patient ZIKV17.

Clock Model	Coalescent Prior	Mean Rate	Median Rate	95% HPD	PS MLE	SS MLE	Bayes Factor (PS)	Bayes Factor (SS)
Strict	Constant	9.88E-04	9.22E-04	2.855E-4 - 1.804E-3	-14,139.86	-14,139.78	-	-
Strict	Skygrid	9.89E-04	9.32E-04	2.651E-4 - 1.786E-3	-14,140.61	-14,140.53	-0.75	-0.76
Strict	Skyline	9.94E-04	9.40E-04	2.999E-4 - 1.805E-3	-14,143.45	-14,143.57	-3.59	-3.80
UCLN	Constant	5.29E-04	5.17E-04	3.109E-4 - 7.612E-4	-14,139.42	-14,139.04	0.45	0.73
UCLN	Skygrid	9.81E-04	9.55E-04	4.835E-4 - 1.576E-3	-14,139.99	-14,139.96	-0.13	-0.19

TS5a- Reference sequences used to design probes.

Sequences (from GenBank) used to design probes for Zika virus targeted enrichment
gi|969945756|gb|KU321639.1| Zika virus strain ZikaSPH2015, complete genome
gi|572167484|gb|KF268948.1| Zika virus isolate ARB13565 polyprotein gene, complete cds
gi|572167486|gb|KF268949.1| Zika virus isolate ARB15076 polyprotein gene, complete cds
gi|572167488|gb|KF268950.1| Zika virus isolate ARB7701 polyprotein gene, complete cds
gi|591399175|gb|KF993678.1| Zika virus strain PLCal_ZV from Canada polyprotein gene, partial cds
gi|685052337|dbj|LC002520.1| Zika virus genomic RNA, complete genome, strain: MR766-NIID
gi|631250742|gb|KJ776791.1| Zika virus strain H/PF/2013 polyprotein gene, complete cds
gi|647734797|gb|KJ634273.1| Zika virus strain CK-ISL 2014 E protein (E) gene, partial cds
gi|592746957|gb|KF383115.1| Zika virus strain ArB1362 polyprotein gene, complete cds
gi|592746959|gb|KF383116.1| Zika virus strain ArD7117 polyprotein gene, complete cds
gi|592746961|gb|KF383117.1| Zika virus strain ArD128000 polyprotein gene, complete cds
gi|592746963|gb|KF383118.1| Zika virus strain ArD157995 polyprotein gene, complete cds
gi|592746965|gb|KF383119.1| Zika virus strain ArD158084 polyprotein gene, complete cds
gi|592746967|gb|KF383120.1| Zika virus strain ArD142623 nonfunctional polyprotein gene, partial sequence
gi|592746968|gb|KF383121.1| Zika virus strain ArD158095 polyprotein gene, partial cds
gi|345132140|gb|HQ234498.1| Zika virus isolate MR_766 polyprotein gene, partial cds
gi|345132142|gb|HQ234499.1| Zika virus isolate P6-740 polyprotein gene, partial cds
gi|345132144|gb|HQ234500.1| Zika virus isolate IbH_30656 polyprotein gene, partial cds
gi|345132146|gb|HQ234501.1| Zika virus isolate ArD_41519 polyprotein gene, partial cds
gi|380036385|gb|JN860885.1| Zika virus isolate FSS13025 polyprotein gene, partial cds
gi|226374362|gb|AY632535.2| Zika virus strain MR 766, complete genome
gi|146411780|gb|DQ859059.1| Zika virus strain MR 766 polyprotein gene, complete cds
gi|258561568|gb|DQ859064.1| Spondweni virus strain SM-6 V-1 polyprotein gene, complete cds
gi|226377833|ref|NC_012532.1| Zika virus, complete genome
gi|189092757|gb|EU545988.1| Zika virus polyprotein gene, complete cds
gi|973447404|gb|KU312312.1| Zika virus isolate Z1106033 polyprotein gene, complete cds

TS5b **Probe** - Probes used for Zika virus targeted enrichment.
file:///Users/danielledurigon/Desktop/TSupplementary%20NEJM%20apr17/TS5.xlsx/Probes.html

TS6- Algorithms for genetic evolution (FEL, MEME and FUBAR).
file:///Users/danielledurigon/Desktop/TSupplementary%20NEJM%20apr17/resources/TS6.html

TS7- GenBank accessions for the consensus genomes–

BankIt2148493	ZKV17Asem_2016-04-26	MH882527
BankIt2148493	ZKV17Auri_2016-04-26	MH882528
BankIt2148493	ZKV17Bsem_2016-05-03	MH882529
BankIt2148493	ZKV17Csem_2016-05-10	MH882530
BankIt2148493	ZKV17Dsem_2016-05-17	MH882531
BankIt2148493	ZKV17Esem_2016-05-24	MH882532
BankIt2148493	ZKV17Fsem_2016-05-31	MH882533
BankIt2148493	ZKV17Gsem_2016-06-08	MH882534
BankIt2148493	ZKV17Hsem_2016-06-16	MH882535
BankIt2148493	ZKV17Jsem_2016-06-28	MH882536
BankIt2148493	ZKV17Ksem_2016-07-05	MH882537
BankIt2148493	ZKV17Lsem_2016-07-12	MH882538
BankIt2148493	ZKV17Msem_2016-07-19	MH882539
BankIt2148493	ZKV17Nsem_2016-07-27	MH882540
BankIt2148493	ZKV17sem_2016-04-19	MH882541
BankIt2148493	ZKV17uri_2016-04-19	MH882542
BankIt2148493	ZKV19Auri_2016-05-05	MH882543
BankIt2148493	ZKV19Bsem_2016-05-12	MH882544
BankIt2148493	ZKV19Csem_2016-05-19	MH882545
BankIt2148493	ZKV19Dsem_2016-05-24	MH882546
BankIt2148493	ZKV19Esem_2016-06-02	MH882547
BankIt2148493	ZKV19Fsem_2016-06-09	MH882548
BankIt2148493	ZKV19uri_2016-04-30	MH882549

4- ADDITIONAL REFERENCES

1. **Baele, G., P. Lemey, T. Bedford, A. Rambaut, M. A. Suchard, and A. V. Alekseyenko.** 2012. Improving the accuracy of demographic and molecular clock model comparison while accommodating phylogenetic uncertainty. Mol Biol Evol **29:**2157-67.
2. **Blackley, D. J., M. R. Wiley, J. T. Ladner, M. Fallah, T. Lo, M. L. Gilbert, C. Gregory, J. D'Ambrozio, S. Coulter, S. Mate, Z. Balogun, J. Kugelman, W. Nwachukwu, K. Prieto, A. Yeiah, F. Amegashie, B. Kearney, M. Wisniewski, J. Saindon, G. Schroth, L. Fakoli, J. W. Diclaro, 2nd, J. H. Kuhn, L. E. Hensley, P. B. Jahrling, U. Stroher, S. T. Nichol, M. Massaquoi, F. Kateh, P. Clement, A. Gasasira, F. Bolay, S. S. Monroe, A. Rambaut, M. Sanchez-Lockhart, A. Scott Laney, T. Nyenswah, A. Christie, and G. Palacios.** 2016. Reduced evolutionary rate in reemerged Ebola virus transmission chains. Sci Adv **2:**e1600378.
3. **Drummond, A. J., S. Y. Ho, M. J. Phillips, and A. Rambaut.** 2006. Relaxed phylogenetics and dating with confidence. PLoS Biol **4:**e88.
4. **Drummond, A. J., G. K. Nicholls, A. G. Rodrigo, and W. Solomon.** 2002. Estimating mutation parameters, population history and genealogy simultaneously from temporally spaced sequence data. Genetics **161:**1307-20.
5. **Drummond, A. J., M. A. Suchard, D. Xie, and A. Rambaut.** 2012. Bayesian phylogenetics with BEAUti and the BEAST 1.7. Mol Biol Evol **29:**1969-73.
6. **Faria, N. R., J. Quick, I. M. Claro, J. Theze, J. G. de Jesus, M. Giovanetti, M. U. G. Kraemer, S. C. Hill, A. Black, A. C. da Costa, L. C. Franco, S. P. Silva, C. H. Wu, J. Raghwani, S. Cauchemez, L. du Plessis, M. P. Verotti, W. K. de Oliveira, E. H. Carmo, G. E. Coelho, A. Santelli, L. C. Vinhal, C. M. Henriques, J. T. Simpson, M. Loose, K. G. Andersen, N. D. Grubaugh, S. Somasekar, C. Y. Chiu, J. E. Munoz-Medina, C. R. Gonzalez-Bonilla, C. F. Arias, L. L. Lewis-Ximenez, S. A. Baylis, A. O. Chieppe, S. F. Aguiar, C. A. Fernandes, P. S. Lemos, B. L. S. Nascimento, H. A. O. Monteiro, I. C. Siqueira, M. G. de Queiroz, T. R. de Souza, J. F. Bezerra, M. R. Lemos, G. F. Pereira, D. Loudal, L. C. Moura, R. Dhalia, R. F. Franca, T. Magalhaes, E. T. Marques, Jr., T. Jaenisch, G. L. Wallau, M. C. de Lima, V. Nascimento, E. M. de Cerqueira, M. M. de Lima, D. L. Mascarenhas, J. P. M. Neto, A. S. Levin, T. R. Tozetto-Mendoza, S. N. Fonseca, M. C. Mendes-Correa, F. P. Milagres, A. Segurado, E. C. Holmes, A. Rambaut, T. Bedford, M. R. T. Nunes, E. C. Sabino, L. C. J. Alcantara, N. J. Loman, and O. G. Pybus.** 2017. Establishment and cryptic transmission of Zika virus in Brazil and the Americas. Nature **546:**406-410.
7. **Garrison, E., and G. Marth.** 2012. Haplotype-based variant detection from short-read sequencing. arXiv preprint arXiv:1207.3907 [q-bio.GN]
8. **Gill, M. S., P. Lemey, N. R. Faria, A. Rambaut, B. Shapiro, and M. A. Suchard.** 2013. Improving Bayesian population dynamics inference: a coalescent-based model for multiple loci. Mol Biol Evol **30:**713-24.
9. **Kingman, J.** 1982. The coalescent. Stoch Proc Appl **13:**235-248.
10. **Lanciotti, R. S., O. L. Kosoy, J. J. Laven, J. O. Velez, A. J. Lambert, A. J. Johnson, S. M. Stanfield, and M. R. Duffy.** 2008. Genetic and serologic properties of Zika virus associated with an epidemic, Yap State, Micronesia, 2007. Emerg Infect Dis **14:**1232-9.
11. **Lemey, P., V. N. Minin, F. Bielejec, S. L. Kosakovsky Pond, and M. A. Suchard.** 2012. A counting renaissance: combining stochastic mapping and empirical Bayes to quickly detect amino acid sites under positive selection. Bioinformatics **28:**3248-56.
12. **Lu, X., X. Li, Z. Mo, F. Jin, B. Wang, H. Zhao, X. Shan, and L. Shi.** 2012. Rapid identification of Chikungunya and Dengue virus by a real-time reverse transcription-loop-mediated isothermal amplification method. Am J Trop Med Hyg **87:**947-53.
13. **O'Brien, J. D., V. N. Minin, and M. A. Suchard.** 2009. Learning to count: robust estimates for labeled distances between molecular sequences. Mol Biol Evol **26:**801-14.
14. **Oliveira, D. B., F. J. Almeida, E. L. Durigon, E. A. Mendes, C. T. Braconi, I. Marchetti, R. Andreata-Santos, M. P. Cunha, R. P. Alves, L. R. Pereira, S. R. Melo, D. F. Neto, F. S. Mesquita, D. B. Araujo, S. R. Favoretto, M. A. Safadi, L. C.**

Ferreira, P. M. Zanotto, V. F. Botosso, and E. N. Berezin. 2016. Prolonged Shedding of Zika Virus Associated with Congenital Infection. N Engl J Med **375:**1202-4.

15. **Park, D. J., G. Dudas, S. Wohl, A. Goba, S. L. Whitmer, K. G. Andersen, R. S. Sealfon, J. T. Ladner, J. R. Kugelman, C. B. Matranga, S. M. Winnicki, J. Qu, S. K. Gire, A. Gladden-Young, S. Jalloh, D. Nosamiefan, N. L. Yozwiak, L. M. Moses, P. P. Jiang, A. E. Lin, S. F. Schaffner, B. Bird, J. Towner, M. Mamoh, M. Gbakie, L. Kanneh, D. Kargbo, J. L. Massally, F. K. Kamara, E. Konuwa, J. Sellu, A. A. Jalloh, I. Mustapha, M. Foday, M. Yillah, B. R. Erickson, T. Sealy, D. Blau, C. Paddock, A. Brault, B. Amman, J. Basile, S. Bearden, J. Belser, E. Bergeron, S. Campbell, A. Chakrabarti, K. Dodd, M. Flint, A. Gibbons, C. Goodman, J. Klena, L. McMullan, L. Morgan, B. Russell, J. Salzer, A. Sanchez, D. Wang, I. Jungreis, C. Tomkins-Tinch, A. Kislyuk, M. F. Lin, S. Chapman, B. MacInnis, A. Matthews, J. Bochicchio, L. E. Hensley, J. H. Kuhn, C. Nusbaum, J. S. Schieffelin, B. W. Birren, M. Forget, S. T. Nichol, G. F. Palacios, D. Ndiaye, C. Happi, S. M. Gevao, M. A. Vandi, B. Kargbo, E. C. Holmes, T. Bedford, A. Gnirke, U. Stroher, A. Rambaut, R. F. Garry, and P. C. Sabeti.** 2016. Ebola Virus Epidemiology, Transmission, and Evolution during Seven Months in Sierra Leone. Cell **161:**1516-26.
16. **Pond, S. L., S. D. Frost, and S. V. Muse.** 2005. HyPhy: hypothesis testing using phylogenies. Bioinformatics **21:**676-9.
17. **Sweredoski, M. J., and P. Baldi.** 2008. PEPITO: improved discontinuous B-cell epitope prediction using multiple distance thresholds and half sphere exposure. Bioinformatics **24:**1459-60.
18. **Wagner, D., K. de With, D. Huzly, F. Hufert, M. Weidmann, S. Breisinger, S. Eppinger, W. V. Kern, and T. M. Bauer.** 2004. Nosocomial acquisition of dengue. Emerg Infect Dis **10:**1872-3.
